# On Extirpation of Fistula in Ano

**Published:** 1855-12

**Authors:** 


On Extirpation of Fistula in Ano. By M. Richard.—In this communication M. Richard has in view only simple cases of fistula, which constitute four-fifths of those met with. Whether an abscess has preceded or not—and its absence is far more common than usually supposed—a fistulous track extends from some part of the circumference of the anus into the rectum, just above the sphincter. The track is nearly straight, without any diverticulum, is usually very short, and easy of examination, providing this be done more gently than is often the case. The symptoms are much the same in all. There is little or no pain in defæcation, unless there has been great constipation, accompanied by painful spasm of the sphincter; and then, as in true fissure of the anus, it is only some minutes after the passage of the stools that the pain is felt. But there is a constant irritation, which is increased by walking, watching, long sitting, and stimulating food. With this is joined itching, from eczema of the orifice. Baths, rest, regimen, touching the anus and the track with concentrated solution of nitrate of silver or tincture of iodine, assuage or remove these annoying symptoms. The linen is stained by two very different fluids—one colorless, sticky, and staining like seamen-spots, is furnished by the eczema or intertrigo; and the other, consisting of prominent, scaly, yellowish spots, is true pus coming from the fistula itself. The patient need not be asked whether the linen is stained with fœcal matters, as in these cases such never traverse the fistula; but we may learn from him that wind escapes involuntarily from the fundament.

Slight as the suffering from this infirmity may seem, an operation should always be advised; for the reported cases of cure are of doubtful authenticity. Iodine and other injections do not succeed in these simple cases, it being a fact which experience alone could have taught, that multiple, long, and sinuous fistulæ are those in which these prove efficacious. The operation M. Richard prefers is that of *extirpation of the track of the fistula.* He has had recourse to it about thirty times, and has always found it innocuous and effective. A grooved director is introduced into the track, the length of which varies from one to two centimeters, and its end is brought out at the anus. This is done very gently, in order to avoid causing pain or laceration of the track, which would prevent the success of the operation. One assistant draws up the buttock forcibly, and another keeps the integuments around the anus as much on the stretch as possible. When the walls of the track are firm, and the folds of the anus well effaced, we may shave along the lower part of the director at a single rapid stroke; but this is a doubtful proceedure, for the bistoury, having its back applied to the anal lip of the sound side, and its edge pressing the internal orifice of the track, may find the latter so soft and depressible as to recede without being cut, and the tissues becoming lacerated during the effort, only an incomplete extirpation results. It is better to plunge the point of the bistoury under the probe to the middle of the track, and having thus fixed the tissues, detach the inner half of the fistulous canal by two or three rapid but well-combined movements, so as to sacrifice as little as possible. Then the external half may be extirpated at one single stroke. Sometimes, when the parts are not kept tense enough, a tenaculum is to be plunged under the track, and the extirpation is practiced at leisure, by cutting from without inwards. In short, the surgeon must extirpate the canal with as little loss as possible, consistent with the complete removal of the pyogenic membrane.

It may be asked, what are the advantages of this operation, which is more difficult to perform and to bear, and leaves a larger wound than that of incision? The advantages are summed up in a word: We are enabled to dispense with tents and all dressings. Tepid fomentations, night and morning, and attention to cleanliness, are all that are required. Two hours after the operation all pain has ceased, and confinement to bed continues only for from twenty-four to forty-eight hours. No accidents or ill consequences result, the wound, in contact with the intestine at one end, and closed in by the margin of the anus at the other, seeming to enjoy all the immunity of subcutaneous wounds. Complete cicatrization may, however, be delayed for even several months, without either patient or surgeon having cause for discouragement.—(*Bul. de Thér.*, tome xlviii. pp. 537–61.) *Medico-Chir. Rev.*

